# A content analysis of actionable guidelines for Climate-Smart agriculture implementation in South Africa- communication for behavioral changes

**DOI:** 10.1016/j.cliser.2025.100541

**Published:** 2025-04

**Authors:** Oladimeji Idowu Oladele, Mjabuliseni Simon C. Ngidi

**Affiliations:** Department of Agricultural Extension & Rural Resources Management, School of Agricultural Sciences, University of KwaZulu-Natal, Pietermaritzburg Campus, King Edward Ave, Scottsville, Pietermaritzburg 3201, South Africa

**Keywords:** Climate change, Advisory services, Risk communication, Content analysis

## Abstract

The actionable guidelines for climate-smart agriculture were developed as advisory and prescriptive activities to promote the adoption of climate-smart agriculture techniques. The coverage of the guidelines aligns with the agroecological principles which serve as the foundation for cleaner production. The content analysis explored the nexus between cleaner production-agroecological principles and climate-smart agriculture, by examining the scope and intensity of coverage of agroecological principles through the perspectives of extended parallel process, construal level theory, and information deficit model by the actionable guidelines. The results reinforce the non-mutual exclusivity and exhaustivity among the three concepts due to the overlapping of the practice and knowledge of the concepts; and that the communication of any of the concepts inadvertently covers the other in a way that coherence, complementarity, and coordination have been established. The communication in the actionable guidelines emphasizes the intention for desired results, current activities needed to be implemented, implementation steps rather than the danger posed by climate change, and future implications and theoretical issues as often with climate change reports and communication outlets. The practical implication of the findings is that communication on climate change should not be overtly scientific if it is to elicit behavioral change and that the efficacy of the communication outlets should be evaluated for effectiveness.

## Introduction

The significant global challenges posed by climate change to humankind are characterized by unknown nature, the unpredictability of its onset, and catastrophic impacts [FAO, 2021]. Climate change affects communities that depend on agro-based livelihoods negatively, posing threats to agriculture, economic growth, and development [Kangah, et al. 2022, Kom et al. 2022]. Adaptation strategies such as the use of early warning systems, agrometeorology information, resilient varieties, water-smart, and soil health technologies are integral components of climate-smart agriculture (CSA) and have been widely promoted over the last decade, as a composite method for sustainability of productivity, incomes, adaptive capacity, and resilience to shocks; to achieve a greenhouse gas emissions reduction, increase carbon sinks, either individually or collectively [Bailey, et al. 2014, Batchelor, et al. 2019]. The forward and backward linkages that exist between agriculture and the economy stress its importance and have been identified as a major sector that could enhance the greening of the overall economy of South Africa [Valle Júnior, et al. 2021]. These linkages are in terms of agriculture being a source and sink of carbon; providing raw materials for the other sector of the economy as well as requiring inputs from industries for its improvement. The agenda of sustainable development goals (SDGs) and the principle of agroecology have emphasized the need for climate action and associated activities.

The prospects of climate change mitigation through agriculture have been through the elimination of emissions along the value chain, direct greenhouse gas emissions from fossil energy, using bioenergy sources, efficient management of carbon and nitrogen elements to reduce emissions, and improving carbon sinks and sequestration. To achieve climate change mitigation, water, energy/carbon, nutrient, weather, and cropsmart practices have been disseminated along the agricultural value chain to reduce GHG emissions. The adoption and improvement of CSA practices increase the propensity for cleaner agricultural production. These practices are often depicted as sustainable agriculture, regenerative agriculture, nature-based solutions, environmentally friendly agriculture, and agricultural clean production technologies. The characteristics of value-chain actors would influence the eco-efficiency and cleaner production decisions on the use of farm equipment and machinery. CSA promotes integrated cleaner production approaches through the minimization of resource extraction, increased use efficiency, recycling of waste residue, and energy savings [Athira, 2019]. Farmers’ adaptation to the challenges of climate change and improving societal well-being is enhanced through the framework of CSA [Zilberman, 2018]. It was indicated that the adoption of CSA among African smallholder farmers is low despite being a frequent issue of discussion among national governments and international policy [Ogunyiola, et al 2022].

Many factors adduced for the low adoption of CSA practices include the lack of coherent, implementable policies at the national and local levels [Sova, et al. 2018]; limited enforcement of policies, and regulations [Ampaire, et al. 2020], and the use of top–down approaches in policy formulation. It was found that effective mainstreaming, coherence, coordination, and integration between various policies, frameworks, and programs dealing with climate change, agricultural development, and food security is a key prerequisite for the adoption and transformation of CSA across Africa [Ifeanyi-Obi, et al. 2022]. Thus, the need to implement inclusive policies and consultation with smallholder farmers to improve the upscaling of CSA [Ogunyiola, et al 2022]; the exclusion of farmers in policymaking processes, science-policy dialogues, and scientific recommendations leads to poor considerations of local practices [Gardezi, et al. 2022]. Where CSA policies already exist, there exists non-functional implementation pathways, indistinct roles of actors, weak linkages among implementing administrative levels, and political interference [Steenwerth, et al. 2014]. To scale up climate-smart technologies and practices, the coordination of relevant policy instruments and institutions to disseminate information, and ensure participation is critical [Ampaire, et al. 2020].

### Climate services

The management of the impacts of land, atmosphere, ocean, and cryosphere phenomenal for the prosperity and productivity of the society is often depicted as climate services (American Meteorological Society, 2023). Climate services cover past, present, and future scenarios emanating from the average weather conditions in the areas of climate data stewardship, analysis, historical summaries, current observations, monitoring, reports, and studies, as well as forecasts, projections for mitigation, planning, and adaptation uses to enhance scalability (Rigby et al., 2022). Similarly, World Meteorological Organization (WMO) (2023) stated that there is a need for tailored climate information and services to address extreme weather, food and water insecurity.

Studies indicate that information is very important, especially accurate and reliable information that supports decisions, and triggers actions that affect the system’s performance (Angello et al., 2016). The components of information worth are information access points which are assessed through the timeliness, adequacy, relevance, and accuracy of information (Meir, 2000). Orgill (2019) stated that effective communication channels facilitate innovation diffusion, thus agricultural extension services play important roles in increasing farmers’ productivity. Kansiime et al. (2021) concluded that there is a need for targeting information to specific users based on household characteristics. Climate information helps smallholder farmers for adaptation to weather variability, thus, information must be accessible, credible, timely, and location-specific and eliminate mismatches of services and users’ needs (Djido et al., 2021; Yegbemey et al., 2021; Tamiru et al., 2022, Kumar et al 2020). Mtega (2021) found that farmers resource endowment determines the information channels used.

Cherotichi et al (2012) stated that radio is the major channel for accessing climate information. Hibbs, et al (2014) stated that agricultural producers in Kansas use the Internet, mobile devices, and radio, as well as print and radio sources, to gain information about climate. Ouedraogo et al. (2021) indicated that radio is the most prominent source of information in Africa and its ownership influences weather and climate decision-making in Africa. According to Mohammed (2013), many farmers preferred radio as an important source of information due to the elimination of the literacy challenges. Yimer (2015) reported that in Southern Ethiopia, radio is widely used to disseminate, technologies, provide market information and announce new varieties.

Climate information services have been leading to an increase in adaptation strategies for climate change, specifically, weather variability (Djido et al., 2021), productivity enhancement, and livelihood protection (Yegbemey et al., 2021, Alidu et al., 2022). The application and use of information in response to risks through anticipatory actions are changing the landscape of its utility, importance, and worthiness. Anticipatory actions help in the reduction, mitigation, and enhancement of impacts of disaster and post-disaster response, through the early warning systems (Wilkinson et al, 2019). Komarek et al (2020) opined that farmers are simultaneously exposed to multiple risks and thus need access to diverse information along the production cycles of their enterprises (Korell et al., 2020). The diversity of farmers’ information needs extends to the contents (Amah et al., 2021); typologies and message adequacy (Kumar et al 2020); alignment to users’ needs (Kumar et al (2020); and preferred sources and channels of information (Mottleb, et al 2017). The majority of research on information needs focussed on production and market risks (Komarek et al 2020), to the neglect of the adequacy of measures required by end-users (Nwafor et al., 2020) specific information for different stages of the value chain (Diemer et al. 2020) and emerging needs (Chen and Lu 2019).

CSA practices enhance the use of cleaner production techniques to improve soil quality, reduce greenhouse gas emissions promote environmentally sustainable development [FAO, 2013], and prevent the production of waste, increasing efficiencies of resource use [Tang, et al 2019]. The processes of cleaner production are sustainable activities related to production, technical, and assessment processes on development, sustainability, consumption, environment, products, and services. The everyday-to-day activities of best environmental practices that reduce natural resource usage, maximize resource usage, output, and sustainability, increase environmental and economic performance, and operational efficiency, minimize waste and emissions, minimize environmental risk, and reduce energy consumption lead to cleaner production; such that topmost in the sustainable development tools are cleaner production and natural resources environmental management systems. The integration of renewable energy with carbon capture and utilization, pollution prevention, and circular economy, are major processes of cleaner production (Vieira and Amaral, 2016).

The emphasis on collective actions by The Intergovernmental Panel for Climate Change (IPCC) stresses the need for agriculture and other land-use sectors to have a synergized approach and response to climate change and adaptation [Höhne, et al. 2017], as well as the development of sectoral policies and programs at national levels [Jiang, et al. 2021, IPCC, 2022] Several policies exist in South Africa on climate change mitigation and are related to collective global actions, the conceptualization of CSA, and stabilization of greenhouse gas (GHG) emissions. Despite the fact that the most elaborate and consultative climate governance systems can be found in South Africa, however, the implementation of national climate plans is obstructed by human and technical capacity, and poor coherence and coordination [Averchenkova, et al. 2019]. Similarly, the mainstreaming of climate change into national policies has gained traction, but policy coherence remains weak across sub-Saharan Africa [England, et al 2018]. CSA policy, plans, and guidelines mainstreaming at the local level are not as strong at the global and national levels [Diko, et al 2021]. It was found that climate change policy implementation has challenges of poor financing, overreliance on funding from the international community, and limited involvement of stakeholders [Mujeyi, et al 2021]; incoherencies exist between policies and legislative documents that address climate change in agriculture, disaster, and risks and food security [Zembe, et al 2021, Bajželj, et al 2014].

CSA embraces interventions on mitigation and adaptation and thus the actionable guidelines for CSA practices implementation in South Africa, to support the country’s transition to an all-inclusive green economy; due to the fact that in cleaner production processes. agriculture offers viable approaches to achieving, adaptation, and mitigation aims [Otto, et al 2018]. Agroecological principles overlap with the concepts and activities in the processes of cleaner production such as recycling, biodiversity, co-creation of knowledge, fairness, connectivity, land, and natural resource governance [HLPE, 2021]. The actionable guidelines support the implementation of evidence-based strategies on farm cleaner agriculture production practices designed for the sustainable transformation of farming systems. The intricacies of the stylized communication patterns on climate change have influenced the adoption and implementation of climate change policies [Verroen, et al. 2013].

The nature and framing of climate change communication suggest that it is under risk communication, which is an exchange of information about risks or hazards among people, and institutions to facilitate solution-orientation. The basic four risk communication theories namely trust determination, negative dominance, mental noise, and risk perception are critical for communication with the public. The theories emphasize that for communication to be effective trust has to be built with the public; risk information and messaging should be short and enhanced with visual aids to enhance recall; avoid negative words and contents and elicit strong concern through presentations of adverse outcomes as involuntary and out of personal control. Climate change has always been presented from the threats and risk angle and thus the need to integrate risk communication into public relations plans.

Similarly related to the risk communication theory is the extended parallel process model (EPPM) which presents climate change information as a threat to existence and livelihoods in order to elicit positive, preventive behavioral changes in the context of existential threat [Trope, et al. 2010]. This describes the combination of efficacy beliefs and fear of threats to determine behavioral decisions. The extended parallel process model is an effective social behavioral change communication theory for protection. In addition to the extended parallel process model (EPPM) is the application of construal level theory (CLT) which states that in planning for the distant future, the understanding of different stakeholders’ views and consideration of alternatives to reality is very important, because forecasts are distinct from direct experience [Van der Linden, 2015, Poortvliet, et al. 2020]. Furthermore, the application of risk communication in climate change has dwelt on the concept of a “deficit model”, to correct fact falsification, more information is required for understanding and behavioral change. Several authors [Branca & Perelli 2020, Mujeyi, et al. 2022, Department of Environment, Forestry and Fisheries 2020] that perceptions of risks associated with climate change and the perception of the capacity to act against the perceived risks are important to stimulate adaptation and mitigation behaviors, however, the contents of communication and the presence, meanings, and relationships of certain words, themes, and concepts would be able to promote the application of cleaner production processes [Hennink, et al. 2011].

This study aimed to evaluate the scope and intensity of coverage of agroecological principles through the perspectives of extended parallel process and construal level theory for the communication of the concepts of cleaner production, agroecological principles, and climate-smart agriculture by the actionable guidelines using content analysis. The study covered specific questions as to what extent are the concepts of agroecology, and cleaner production reflected in the actionable guidelines and to what extent the actionable guidelines include elements of efficacy, current intentions, and practicality as often with climate change reports and communication outlets.

## Materials and Methods

This paper analyzed the actionable guidelines for CSA implementation in South Africa to examine the presence, meanings, and relationships of certain words, themes, and concepts on cleaner production. This is predicated on the fact that there is a lack of practical guidelines for the implementation of the considerable body of knowledge on CSA that exists in South Africa. The Department of Environment, Forestry, and Fisheries released the climate-smart framework to the public with the “objectives of guiding actions at all levels on main-streaming CSA plans, programs and projects; contributing to increased productivity and growth of value chains; enhancing resilience to climatic shocks; contributing to reducing national emission intensity and for effective implementation of the CSA across all levels [FAO 2012]. The actionable guidelines were released in two volumes namely: Actionable guidelines for the implementation of CSA in South Africa. Volume 1: “Situation Analysis and Actionable Guidelines for the Implementation of CSA in South Africa. Volume 2: Climate Smart Agriculture Practices” with 201 pages with 98,721 words and 106 pages with 46,091 words respectively. The two volumes provide detailed explanations and practical steps for the implementation of CSA practices by farmers, extension agents, and policymakers to facilitate the country’s transition to a green economy, dissemination of agricultural technologies to farmers, and implementation of CSA in South Africa. The two volumes were organized into sections on soil, and water management, cereal-based cropping systems, Sugar cane production, fruit production and viticulture, climate information services, Weather index-based insurance, urban agriculture, management of rangelands/pasturelands, agro-processing, marketing, knowledge dissemination, gender and social inclusion, and policy issues.

The two volumes’ content was analyzed by coding the text in the reports using the concepts from cleaner production processes, such that directed content analysis was carried out for each of the concepts. The codes applied in the content analysis are efficiency, green, sustainable, mitigation, recycling, soil health, input, diversification, synergy, circular, ecology, diversity, biomass, wastes, knowledge, governance, participation, connectivity, fairness, social, natural, and animal health. Sentences or text segments that were identified as addressing a concept from one of the cleaner processes were labeled with codes. Each of the concepts associated with cleaner production was thus independently used as codes which were counted to ascertain the frequency of occurrences of each code used, resulting in a total frequency for each concept thus making the analysis deductive and quantitative [PRI, 2020], and focused on the frequency and timing of concepts in the report. The occurrence of the codes in relation to cleaner production in different sections of the two volumes to deduce the meanings, and relationships of certain words, themes, and concepts. To explore the dimensions of communication theories, namely the extended parallel process model (EPPM), Construal level theory (CLT), and deficit model, a general coding was applied that cut across the risk communication theories as susceptibility which encompasses fear and threat and response covering for the dimensions of efficacy. This general coding was to determine the extent to which the Actionable guidelines for the implementation of CSA in South Africa presented and applied susceptibility and response concepts in relation to cleaner production. EPPM was coded as Threat and efficacy, CLT as future and current, and deficit model as practical and not practiced.

## Results

**Fig. 1:**
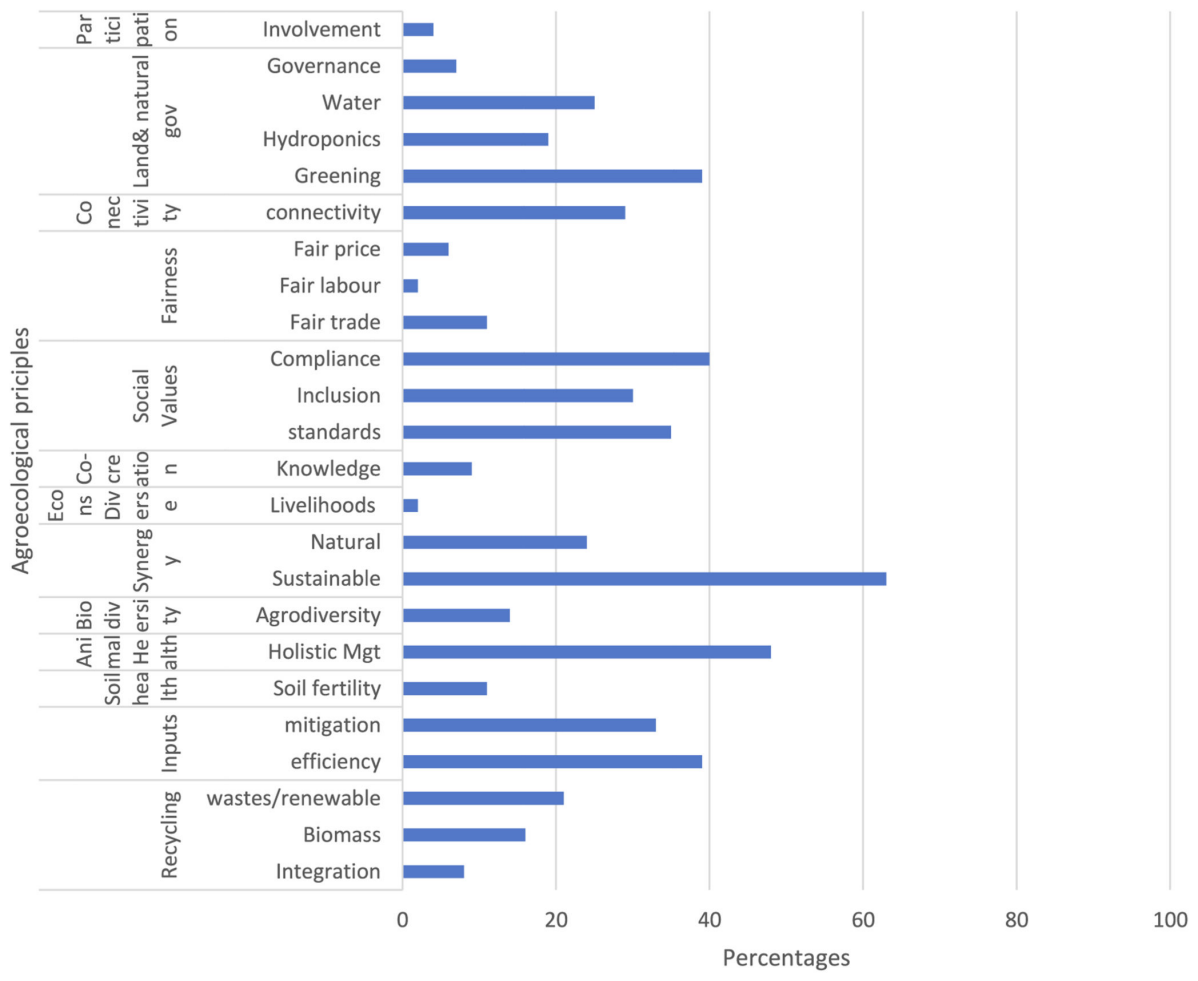
Frequency of cleaner production concepts relative to agroecological principles.

**Fig. 2:**
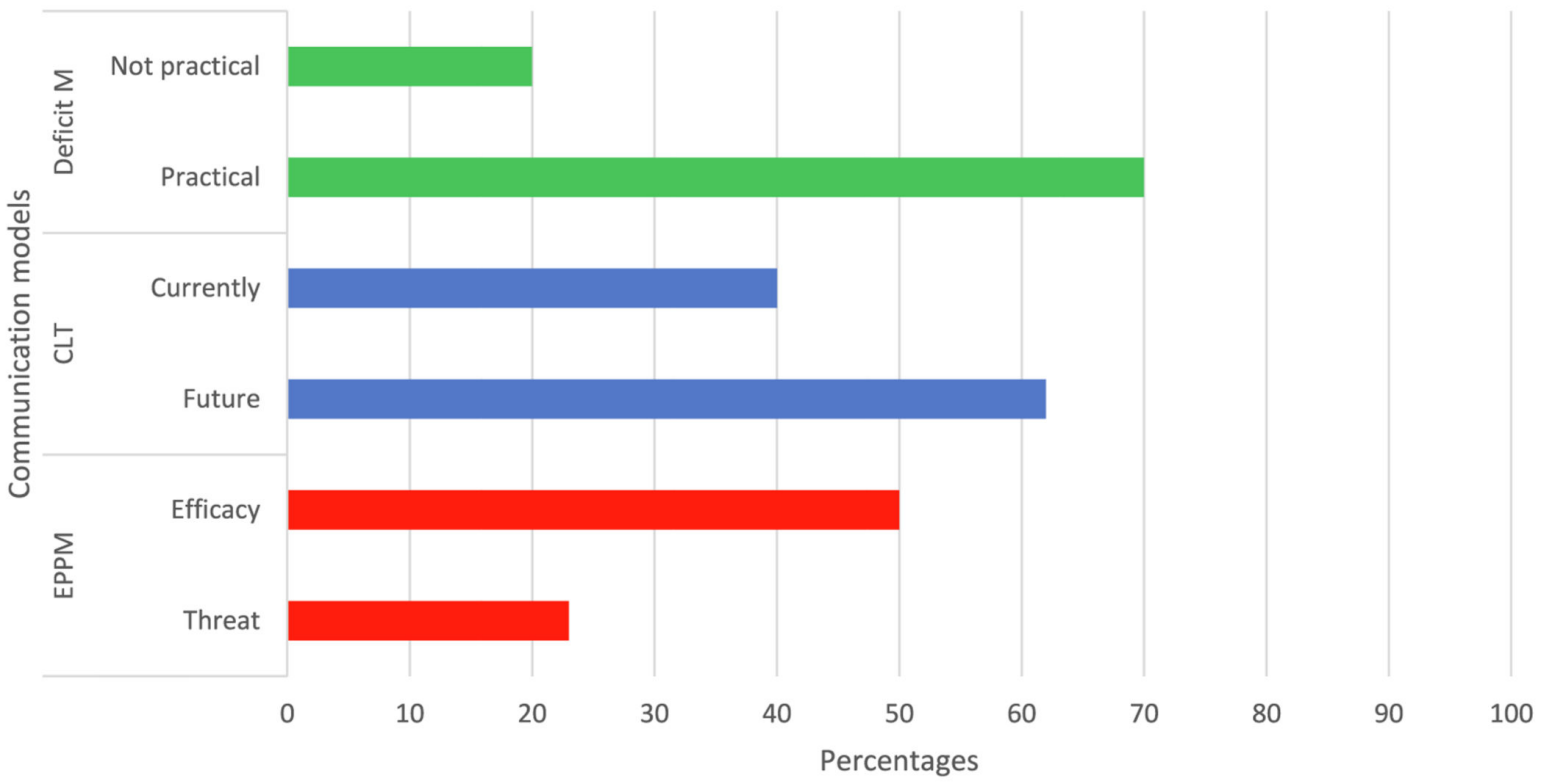
Frequencies of extended parrallel model, construal level theory and deficit model in actionable guidelines.

The results presented in Figs. 1 and 2 show the frequency and percentages of the indicators used in the analysis of the coverage of actionable guidelines on CSA in South Africa and the narratives associated with each of the indicators. This paper examined the coverage of concepts and the presentation of cleaner production communication in the actionable guidelines on CSA reports in South Africa. This is guided by underpinning principles of agroecology and theoretical frameworks of the extended parallel process model (EPPM), Construal level theory (CLT), and deficit model which were coded as threat & efficacy; future & current and practical & impractical respectively. The results section is organized into two sections namely; cleaner production communication and risks communication through actionable guidelines on CSA. Fig. 1 presents the percentages of codes related to cleaner production based on the agroecology principles, while Fig. 2 covers the indicators of the communication models applied in the documents.

### Communication forms through actionable guidelines on CSA

Fig. 1 displays the frequency of use of cleaner production concepts in the actionable guidelines on CSA.

## Discussion

The discussions are organized into two sections namely cleaner production communication through actionable guidelines on CSA and risk communication through actionable guidelines on CSA.

### Greening

The content analysis of actionable guidelines on CSA captures green as a key cleaner production concept, and covers greening, GHG minimization, greenhouse/hydroponics, green water, and green manure; with a total of 85 counts. The creation of prosperity for all people through a green economy ensures that practices such as green economy, eco-innovation, green technologies, and low carbon footprints, have been introduced to promote cleaner production practices and enhance sustainable development and environmentally friendly models. The green economy is people-centered, promotes generational equity, safeguards nature, and supports low-carbon, resource-conserving, diverse, and circular consumption and production; which overlaps with agroecology practices in the use of natural resources and priorities of local farmers. Greening economy with agriculture promotes the application of an ecosystem approach to agriculture, and related fields to address multiple societal needs and desires, for current and future generations [Gallego-Schmid, et al. 2020].

### Fairness

The content analysis of actionable guidelines on CSA captures fairness as a key cleaner production concept and covers fair labor practices, fair price, and fair trade, totaling 19 counts. The guidelines emphasized that CSA practices should enhance dignified and robust livelihoods for food systems actors, such that actors and communities are empowered and take control of their agricultural production, decision-making process resource use, and livelihoods. Similarly, the actionable guidelines iterated that fairness practices include codes of conduct, accountability, risk assessment, collaboration, Sourcing and supplier relations, and monitoring [Fair Trade International, 2023]. The actionable guidelines proffer systems’ orientation to enhance CSA practices. This is in congruence with the collective actions as stated in agroecological principles. Closely related to fair labor practices is fair trade, which increases value-chain actors’ voice, influence, access to markets, finance, subsidies, land rights, and fair value for their products [Gallego-Schmid, et al 2020]. Fairness in cleaner production processes emphasizes independence in procedures, transparency of standards, unbiased, representative, non-discriminatory, and consistency with contractual terms for decision-making.

### Sustainability

The content analysis of actionable guidelines on CSA captures sustainability as a key cleaner production concept, and covers agricultural practices, value chains, sugar production, fruit farming, natural resource management, water use/irrigation, urban agriculture, renewable energy, and agro-processing, with a total of 65 counts. Sustainability is captured in relation to the balancing of the need for food production and the preservation of environmental ecosystems, by promoting healthy biodiversity management of natural resources, and economic viability, in addition to social and economic equity.

### Mitigation

The content analysis of actionable guidelines on CSA capture mitigation 82 times as a key cleaner production concept, and covered interventions, strategies, options, resilience, carbon sequestration, climate-smart pillars, HRLM, and holistic planned grazing. The use of cover crops and green manures increases soil carbon sequestration, and fertility, mitigating climate change, and improving crop productivity. The actionable guidelines on CSA stress slowing and closing resource solutions as means of mitigation. Slowing, closing, and narrowing resource loops prevent and reduce GHG emissions [Akhtar, 2020]. Cleaner production reduces hazards, pollution, emissions, waste generation, and environmental degradation, and maximizes renewable energy [Bhattacharyya, et al. 2021].

### Recycling

The content analysis of actionable guidelines on CSA captured recycling as a key cleaner production concept 15 times, and covers grey water, food wastes, wastewater, composting, and animal manure. Recycling can be closed-loop, on-site, re-use, off-site, and reclamation to derive the benefits of cleaner production by eliminating costs associated with waste handling, insurance premiums, and Health Safety Environment (HSE) damage costs.

### Soil health

The content analysis of actionable guidelines on CSA captures soil health as a key cleaner production concept 12 times, and covers restoration, integrated soil fertility management, reduced use of chemical fertilizer, conservation agriculture, and zero and minimum tillage. To ensure cleaner production, some activities associated with soil health include the use of organic compost and biofertilizers [Soobhany, 2019]. Vermicompost is ranked as the best fertilizer, due to its humic substances and microbiologically active ensuring a greener and cleaner process that involves bio-oxidation and stabilization [Gupta, et al. 2009] to offset the detrimental environmental impacts of synthetic agrochemicals [Fet, 2023].

### Inputs

The content analysis of actionable guidelines on CSA capture input as a key cleaner production concept, off-farm inputs, low-cost inputs, organic and inorganic inputs, labor inputs, yield potential, and CSA pillars with a total of 40 counts. The cleaner production process ensures that inputs are processed, recycled, and reused, while outputs reduce waste and emissions [Tsangas, et al, 2020]. The actionable guidelines on CSA recommend and highlight several ways inputs can be processed, recycled, and reused along the value-chain processes. Eco-efficiency in agriculture as a cleaner production technique addresses all aspects of inputs, production, and outputs. The agriculture sector plays a crucial role in green, circular, and bio-economy for the main-streaming of global sustainability concepts [Jimenez-Lopez, et al. 2020]. The transformation of the agri-sector into a circular is critical due to the fact that it produces a high percentage of biomass [D’Adamo, et al 2020], which constitutes a critical aspect of the bio-economy [Sinclair, 2017].

### Efficiency

The content analysis of actionable guidelines on CSA captures efficiency as a key cleaner production concept totaling 74 counts. The actionable guidelines on CSA covered efficiency in terms of fertilizer efficiency, irrigation efficiency, water use efficiency, input use efficiency, and crop residue efficiency. Efficiency relates outputs to inputs, such that increasing the efficiency ratio of an input has often been associated with the reduction of other inputs [Singh, et al. 2018]; thus, leading to the reduction and the elimination of dependency on costly, scarce, and environmentally damaging purchased inputs to increase self-sufficiency. Efficiency is closely related to recycling, using co-existing microbiomes to improve nutrient uptake, resilience, and defenses against attacks [Renard, et al. 2023]. The combination of two or more components in a production system increases output than monocultures through an integrated system of biotic and abiotic diversity and alignment in time and space. Findings from studies on Land Equivalent Ratios (LER) have confirmed this assertion.

### Diversity

The content analysis of actionable guidelines on CSA captures diversity as a key cleaner production concept 15 times, and covers diet diversity, agroforestry, and organic farming. To achieve biodiversity the actionable guidelines on CSA explore activities and practices to achieve and protect biodiversity in the crop, and livestock agro-value-chains. Specific references were made to sugarcane production as well as the processing and packaging of agro-produce. Crop diversity safeguards the impact of droughts and high temperatures on food production and crop biodiversity leading to greater resilience to weather [Brooker, et al. 2021]. Agricultural sustainability is enhanced by positive biodiversity–ecosystem function (BEF) effects, biodiversity preservation, improved soil health, reduced greenhouse gas emissions, and reduced use of synthetic inputs [Dawson, et al. 2020]. A high degree of diversity among species improves productivity through vital ecosystem services provisioning [WWF, 2021, Deaconu, 2021]. Positive relationships between agrobiodiversity and increased dietary diversity for farmers in Kenya, Tanzania, Malawi, India, Bolivia, and Guatemala were reported [Madsen, 2012, Santoso 2023, Frison, & Clément, 2020]. The actionable guidelines on CSA depicted dietary diversity, consumption of culturally significant foods, and reduced exposure to toxic synthetic agrochemicals as benefits associated with diversity, and social values of climate-smart adaptation [FAO, 2022].

### Knowledge

The content analysis of actionable guidelines on CSA captured knowledge as a key cleaner production concept 10 times, and covered practical knowledge, dissemination, experiential, and indigenous, knowledge. According to [Glassman, et al. 2018, IPES & ETC Group 2021], agroecology requires intensive knowledge, of environment, culture, and social relevance within the grassroots paradigm that empowers people and communities with the agency of change for sustainable rural development. The multi-stakeholder perspectives emphasize that co-created agricultural knowledge enhances sustainable outcomes. Knowledge co-creation and knowledge co-production serve as an effective strategy to link diverse types of knowledge, among value-chain actors, and foster participatory learning and development which had existed among value-chain actors and throughout communities based on interpretation of understanding, and application. The co-production of knowledge would help to reverse the depletion of natural resources, and widening inequalities, as a shared human endeavor with the collective right to participate [Kansanga, et al. 2019]. In Malawi farmer-to-farmer knowledge co-creation significantly improved social networks and social capital, the exchange of farm products within communities, and building social ties in Ecuador and Uruguay [Deaconu, et al 2019, Bezner-Kerr et al 2022].

### Social

The content analysis of actionable guidelines on CSA captures social values as a key cleaner production concept totaling 106 counts and covers standards, inclusion, responsibility, performance, characteristics, benefits, innovation, compliance, and ecology. The actionable guidelines on CSA place a strong emphasis on human and social values. Social values such as rights in relation to humans, the environment, sustainability, and animal welfare in ethical consumption and sustainable development goals are socially driven agendas that reflect social values and trajectories. Agroecology practices enhance social well-being, livelihoods, meaningful work, and gender and social equity [Barrios et al 2020]. The actionable guidelines on climate-smart agriculture emphasized the repositioning of food systems to be reactive to societal demands in a sustainable manner [Wezel, et al 2020, Steinhäuser, 2020]. Ethnic communities in mountainous landscapes in Italy and Argentina explored Indigenous cultural values to manage and sustain agroecological practices, despite pressures from tourism and outmigration [Wang, et al. 2019].

### Animal health

The content analysis of actionable guidelines on climate-smart agriculture captures knowledge as a key cleaner production concept with a total of 49 counts, and covers manure, commercial farming, transgenic, rangeland management, nutrition, welfare, and HLMR. The actionable guidelines on climate-smart agriculture stressed the need to reshape livestock systems and adapt them into agro-ecosystems for improved natural resources management and ecosystem services provisioning. Similarly, practices for integrating livestock systems into climate-smart agriculture were listed as practices to improve animal health; decrease input use; reduce emissions; enhance diversity; and preserve biodiversity. This is predicated on the fact that the ways animals are incorporated into food systems determine the consequent environmental problems. The actionable guidelines on CSA listed operational steps for planned biodiversity and nature conservation through crop-livestock integration and a recoupling of nutrient cycles.

#### Risk communication through actionable guidelines on CSA

##### Threat and Efficacy

The content analysis of actionable guidelines on CSA revealed that threat-related messages were coded 23 times. The guidelines expatiated threats in terms of the need for conservation agriculture, CSA, sustainability of resource use, pests, diseases, and invasive plants. The guidelines also covered areas related to the susceptibility of farmers to climate change, low productivity, and resilience of holistic livestock resource management. Fifty sections of the actionable guidelines on CSA were coded as efficacy. The efficacy section covered the response plan, adaptation strategies, climate information services, weather index-based insurance, capacity development, gender mainstreaming, policies, and initiatives. The list of activities and practices proffered in the guidelines were to promote response to integrated soil fertility management, increased soil carbon sequestration, yield response, and reduced fossil fuel consumption. Similarly, policies and initiatives in relation to National Climate change response, land care programs, food aid, and safety nets. All these underscore the actions that can be taken to promote cleaner production processes. Severally, the need for actions on mitigation and adaptation and how to ensure their implementation were enumerated.

The perception of the likelihood of the risks associated with climate change would enhance engagement in pro-environmental behaviors such as cleaner production and that the views and actions may not always complement each other [Brügger & Pidgeon 2018]. The link between perception and actions to support climate action provides insights into ways in which climate change communicators can articulate positive perceptions and attitudes to activate effective behavioral responses. Climate change is an area in which perception and actions may operate independently rather than interdependently [Duan, et al. 2022]. The perceived outlook of climate change images leads to greater mitigation behavioral intentions, but the perception may change when the actions of other role players are considered [Chen, 2020]. Ecological world-views are positively related to the perception of the occurrence of climate change and environmental concerns and motivate pro-environmental behaviors [Fragnière, 2016].

##### Future and current intentions

From the actionable guidelines on CSA, references to the future as a consequence of climate risks were coded 65 times, and the guidelines reflected future intentions, sustainable future, adaptation, scenarios, climate services, food demand, outcomes, and services. The guidelines also stated the need to discontinue current practices that are not promoting sustainable practices nor cleaner production such as poor involvement of local participation, extractive processes of natural resources, and the neglect of soil health as well as poor livestock management practices. Climate change is often regarded as a threat that does not cause immediate, direct negative impacts on any single person alone; but understanding threats at personal and societal levels increases the knowledge of the effects of perceived threats on promoting pro-environmental intentions. The general perception of climate change is a distant threat and that the consequences manifest in other places, consequent on others, and are often classified as the government’s responsibility [Schuldt, et al 2018, Ma, et al. 2023]. Individuals with a high perception of climate change as a personal threat reported the highest intention to act to mitigate the effects of climate change [Bostrom, et al 2019]. Climate change as a collective event threatens and elicits efficacy at the societal or collective level because it affects a large number of people and requires collective efforts for mitigation [Wang, 2023]. The knowledge of the susceptibility to threat positively affects the acceptance of carbon offset programs and the achievement of carbon neutrality goals among managers and policymakers.

##### Practical and not practical

The content analysis of actionable guidelines on CSA revealed that the guidelines deviated from the “deficit model”, through the presentation of clear information to elicit behavioral change and including practical steps to achieve CSA and cleaner processes. The practical messages were coded 23 times in terms of specific preparedness to enhance understanding and behavioral change. Climate change as disaster information must be accessible, comprehensive, and targeted to needs to influence preparedness [Abunyewah, et al 2020]. The actionable guidelines on CSA did not present scientific knowledge as superior to public knowledge, nor enforce a top-down approach to communication, rather considered the contexts for cultural, social, and value systems in which the guidelines would be applied [Stoutenborough, & Vedlitz 2014]. Climate change communication should be a constitutive process of producing shared meanings because information provision is important but not sufficient to create behavioral engagement [Ballantyne, 2016].

## Limitations of the study

The study covers only the concepts related to cleaner production from the CSA actionable guidelines and their implications for cleaner production, thus the communication of CSA practices also covers cleaner production practices. The concepts identified from the content analysis were organized according to the agroecology principles due to the global dominance of these principles in CSA and cleaner production approaches [Akamani, 2021]. The categorization of extended parallel process, construal level theory, and information deficit model as reflected in the results are based on a-priori experiences. It is important to note that the application of the communication theories and models in this study was due to the fact that the focus was not on the formation of perception but rather the analysis of the content of climate communication.

### Practical implications

One of the major climate actions as stated by the United Nations is that communication on climate change should educate and mobilize audiences for actions against climate crisis through the use of use authoritative scientific information, conveying the problem and the solutions, and mobilization of actions (UN, 2023). Communication on climate change must therefore reflect the empowerment of the people, distributive justice, and elimination of greenwashing and stereotypes. Depoux et al (2017) affirm that communication of climate change should provoke response individually and collectively to climate change rather than the magnification of threats, fear, and risks associated with its impacts. The juxtaposition of mass media, climate change science, and policy is central and dynamic in the field of communication studies in attempts to shape public perception of global climate change. The use of the right words, language, and framing would therefore affect the message being conveyed and the consequent behavioral changes to the issues of climate change. This implies that policymakers, scientists, and communicators should explore communication transmission and public understanding models in relation to expert knowledge and ‘lay knowledge’.

## Conclusions

This study investigated the communication of cleaner production among farmers, extension, and policymakers through actionable guidelines for CSA implementation in South Africa through content analysis. The study covered specific questions as to what extent are the concepts of agroecology, and cleaner production reflected in the actionable guidelines and to what extent the actionable guidelines include elements of efficacy, current intentions, and practicality as often with climate change reports and communication outlets. It showed that the actionable guidelines promoted cleaner production practices through concepts such as greening, fairness, sustainability, mitigation, recycling, soil health, inputs, efficiency, diversity, knowledge, and social and animal health. From the perspectives of extended parallel process and construal level theory for the communication of the concepts of cleaner production, agroecological principles, and climate-smart agriculture by the actionable guidelines using content analysis. The actionable guidelines deviated from the usual communication on climate change by projecting efficacy, current intentions, and practical activities rather than threat, future, and non-practice as often with climate change reports and communication outlets. The use of guidelines and policies to enhance the adoption of climate-smart practices needs to iterate practical steps for present actions in a practicable way, similarly, communication efforts, on climate change risk communication should be able to project information that elicits behavioral change towards cleaner production processes. The Intergovernmental Panel on Climate Change summary for policymakers contained information elements of threat and not efficacy. The practical implication of the findings is that communication on climate change should not be overtly scientific if it is to elicit behavioral change and that the efficacy of the communication outlets should be evaluated for effectiveness.

## Supplementary Material

Corrigendum

## Data Availability

Data will be made available on request.
