# Supplementary material for: A content analysis of actionable guidelines for Climate-Smart agriculture implementation in South Africa- communication for behavioral changes
Source: Clim Serv. Author manuscript; Available in PMC 2026 Feb 24. (PMC7618795; doi:10.1016/j.cliser.2025.100541)
Supplement: Corrigendum [file EMS212477-supplement-Corrigendum.pdf]

Climate Services 39 (2025) 100576

Contents lists available at ScienceDirect

Climate Services

journal homepage: www.elsevier.com/locate/cliser

Corrigendum

Corrigendum to "A content analysis of actionable guidelines for Climate-Smart agriculture implementation in South Africa- communication for behavioral changes" [Clim. Serv. 38 (2025) 100541]

Oladimeji Idowu Oladele *, Mjabuliseni Simon C. Ngidi

Department of Agricultural Extension & Rural Resources Management, School of Agricultural Sciences, University of KwaZulu-Natal, Pietermaritzburg Campus, King Edward Ave, Scottsville, Pietermaritzburg 3201, South Africa

The authors regret that **Funding**: This research was partially funded by the Welcome Trust's Climate and Health Programme as part of the Sustainable and Healthy Food Systems—Southern Africa (SHEFS-SA) Project (grant number: 227749/Z/23/Z). For the purpose of open access, the authors have applied for a CC BY public copyright license to any author-accepted manuscript version arising from this submission.

The authors would like to apologise for any inconvenience caused.

DOI of original article: https://doi.org/10.1016/j.cliser.2025.100541.

* Corresponding author.

E-mail addresses: oladeleo@ukzn.ac.za (O.I. Oladele), ngidim@ukzn.ac.za (M.S.C. Ngidi).

https://doi.org/10.1016/j.cliser.2025.100576

Available online 10 May 2025

2405-8807/© 2025 The Author(s). Published by Elsevier B.V. This is an open access article under the CC BY license (http://creativecommons.org/licenses/by/4.0/).
